# Microstructure and Efflorescence Resistance of Metakaolin Geopolymer Modified by 5A Zeolite

**DOI:** 10.3390/ma16227243

**Published:** 2023-11-20

**Authors:** Yuwei Lu, Luxia Song, Yuan Xu, Ping Duan, Xiaoming Wang

**Affiliations:** 1 Faculty of Materials Science and Chemistry, China University of Geosciences, Wuhan 430074, China; 2 College of Architecture and Material Engineering, Hubei University of Education, Wuhan 430205, China; 3 Institute for the Application of Green Energy Material, Hubei University of Education, Wuhan 430205, China; 4 Zhongxiang Municipal Transportation Bureau, Zhongxiang 431900, China

**Keywords:** 5A zeolite, metakaolin, cation exchange, efflorescence resistance

## Abstract

In order to reduce the degree of efflorescence in alkali-activated metakaolin geopolymers, a modified 5A zeolite with cation-exchange properties was used to reduce the content of free alkali metal cations in the geopolymer. This work aims to investigate the effect of different dosages of modified 5A zeolite on the microstructure and properties of geopolymer by using compressive strength testing, pore structure analysis (BET), and SEM-EDS. The cation content in the leachate was evaluated using inductively coupled plasma atomic emission spectrometry (ICP-OES). The efflorescence area of the geopolymer was calculated using Image Pro Plus (IPP) software to evaluate the effect of modified 5A zeolite on the degree of efflorescence of the geopolymer and to reveal the effect of modified 5A zeolite on the migration patterns of Na^+^ and Ca^2+^ in the geopolymer. The results showed that modified 5A zeolite with a 4 wt.% content could optimize the pore structure and enhance the mechanical properties of MK geopolymer through internal curing and micro-aggregate effects, which could also exchange cations with the pore solution to form (N, C)-A-S-H gels. The Na^+^ leaching was reduced by 19.4%, and the efflorescence area of the MK geopolymer was reduced by 57.3%.

## 1. Introduction

As an alternative to Portland-cement-based materials, alkali-activated cementitious material is widely used in the construction industry for its excellent early strength, good acid resistance, low permeability, and environment-friendly properties [1,2,3]. The alkali-activated material with low Ca content in the system (Ca/(Si + Al) < 1) is also known as geopolymer [4]. As a new, green, and sustainable building material with low energy consumption, geopolymer with an amorphous three-dimensional network structure is usually formed by geopolymerization reactions between alkali activators and aluminosilicate materials under alkaline conditions [5].

However, both conventional and new cementitious materials suffer from the problem of efflorescence. During the geopolymerization reaction, excess alkali metals in the alkaline activator are mainly present in the form of hydrated cations in the pore solution or are adsorbed on the gel surface. In fact, under a long-term exposure to the external environment, a large number of free alkali metal ions in the matrix tend to carbonate with CO_2_ in the outer environment to form carbonate deposits. The crystallization pressure of crystalline products due to efflorescence deteriorates mechanical properties and affects durability [6,7]. In particular, the efflorescence can cause the degradation of N-A-S-H/N-(C)-A-S-H gels in geopolymers as reported by Srinivasamurthy et al. [8].

Many scholars have tried various methods to reduce the degree of efflorescence. Cappelletti et al. [9] used hydrophobic silica-based materials to modify lime mortar and placed it in an exposed environment. It could be observed that the quantity of crystalline salts on the surface was reduced. Maghsoodloorad et al. [10] found that different types of alkali activators affected the degree of efflorescence in phosphate cement. As the radius of potassium atoms is greater than that of sodium atoms, the internal Na^+^ was more likely to diffuse through the pores to form efflorescence products on the surface. Zhang et al. [11] discussed the relationship between the composition of fly-ash-based geopolymer and its pore structure and efflorescence; they noted that the rate of efflorescence depended on the activation conditions, slag addition, and curing temperature. Márlon A. Longhi et al. [12] optimized the geopolymer ratio by adjusting the synthesis parameters to increase the quantity of soluble silicates in the system to inhibit efflorescence. The reduction in efflorescence and porosity was associated with a high content of Si in the geopolymer. The leaching of alkali metal ions was also retarded. Srinivasamurthy et al. [8] showed that the addition of an appropriate amount of slag to fly ash geopolymer increased the formation of C-A-S-H gels to capture free Na^+^ in the matrix and reduced the porosity to inhibit efflorescence. Gao et al. [13] found that geopolymer with a 1% nano-SiO_2_ content had higher strength, higher density, and lower porosity. The filling effect of nano-SiO_2_ and the gels produced at later reaction were found to be the main reasons for efflorescence resistance. Generally, an excessive amount of activator is required to increase the degree of geopolymerization [14]. However, higher alkalinity leads to a higher free alkali metal ion content. An optimal ratio that achieves the lowest degree of efflorescence needs to be determined [15]. In summary, the best strategy to inhibit efflorescence is to reduce the number of free alkali metal ions and optimize the porosity of the system to reduce the leaching of alkali metal cations.

Most of the current research focuses on optimizing the pore size distribution of geopolymers and modifying the hydrophobic surface to reduce the leaching of alkali metal ions. Some minerals have been found to have excellent cation-exchange properties due to their unique structure. The framework structure of zeolite consists of [Si-O_4_]^4−^ and [Al-O_4_]^5−^ tetrahedral units [16], which are connected in various ways to form a mesh skeleton structure and have a high thermal and hydrothermal stability. The cavities and pores in zeolite cause it to have a large specific surface area [17]. The Al atoms in the skeleton make the basic units negatively charged; to maintain electrical neutrality, positively charged metal cations must be introduced, which can be exchanged with each other, and the exchangeable cations may vary according to the type of zeolite. Theoretically, the higher the Al content in the zeolite skeleton, the higher the cation content and the ion-exchange capacity of the zeolite. And zeolites are often used as molecular sieves in a wide range of applications such as water desalination [18], materials for detergents [19], and disposal of radioactive waste [20]. Zeolites have also been employed in the field of adsorption because of their huge specific surface area. The 5A zeolite used in this study has a cubic crystal structure, which is obtained by ion exchange of Na^+^ with Ca^2+^. The 5A zeolite has a pore size of 4.8 Å and a low Si/Al ratio with a good cation-exchange property. This work proposes to introduce modified 5A zeolite as a cation-exchange material into the geopolymer to reduce the content of the alkali metal Na^+^ in solution and investigate the microstructure and anti-efflorescence property of the modified 5A zeolite–metakaolin geopolymer with the aim of inhibiting efflorescence. The results of this study can provide a new way to improve the apparent quality of geopolymers and provide a theoretical basis for the design, preparation, and application of highly durable geopolymers.

## 2. Materials and Methods

### 2.1. Raw Materials

Water glass was obtained from the Guangdong Foshan Zhongfa Water Glass Factory, containing 9.87% Na_2_O, 32.95% SiO_2_, and 57% H_2_O with a SiO_2_/Na_2_O ratio of 3.45. The sodium hydroxide used to adjust the water glass modulus was supplied by Sinopharm Chemical Reagents Ltd., and the purity was more than 99%.

The 5A zeolite powder (ZT) was purchased from XinTao Technology Co., Ltd. (Pingxiang, China) and the metakaolin powder (MK) was purchased from TianHong Mining Co., Ltd. (Kunming, China). The chemical compositions of MK and ZT are shown in Table 1.

**Figure 1:**
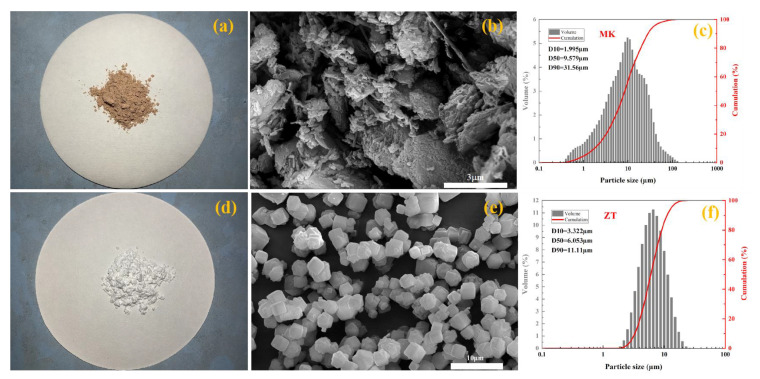
Physical properties of raw materials: (**a**) image of MK, (**b**) microstructure of MK, (**c**) particle size distribution of MK, (**d**) image of ZT, (**e**) microstructure of ZT, and (**f**) particle size distribution of ZT.

Figure 1a,d show the images of MK and ZT, respectively, and Figure 1b,e show the microstructure of MK and ZT, respectively. MK appears as a multi-layered stacked structure, and ZT shows a regular cubic shape.

The particle size distributions of the two raw materials are shown in Figure 1c,f, with the size range of MK being d10–d90: 1.995–31.56 μm and the size range of ZT being d10–d90: 3.322–11.11 μm.

### 2.2. Experimental Procedure

#### 2.2.1. Preparation of Activators

A lower water glass modulus (SiO_2_/Na_2_O molar ratio) can increase the proportion of oligomeric silicone tetrahedral groups to facilitate the synthesis of geopolymers [21]. As previous studies have shown, an activator with a modulus of 1.5 is more effective in stimulating MK activity [22]. In this experiment, the activator was a mixture of NaOH and water glass with a modulus of 1.5. After being stirred and sealed, the activator was left to stand for 24 h. The amount of NaOH to be added was calculated using Equation (1):

(1) $$m_{NaOH}=2\times M_{NaOH}\left(\frac{W_{{SiO}_{2}}\times m_{{Na}_{2}O\cdot {nSiO}_{2}}}{M_{{SiO}_{2}}\times 1.5}-\frac{W_{{Na}_{2}O}\times m_{{Na}_{2}O\cdot {nSiO}_{2}}}{M_{{Na}_{2}O}}\right)$$

where *m_NaOH_* is the mass of added NaOH; *m_NaOH_* is the molecular weight of NaOH; *m_Na_*_2*O·nSiO*2_ is the mass of added water glass; *W_SiO_*_2_ and *W_Na_*_2*O*_ are the mass fractions of SiO_2_ and Na_2_O in water glass, respectively; and *M_SiO_*_2_ and *M_Na_*_2*O*_ are the molecular weights of SiO_2_ and Na_2_O, respectively.

#### 2.2.2. Modification of 5A Zeolite

The structure and components of zeolite can be changed through high-temperature modification. Therefore, on the basis of thermal activation treatment for a wide range of mineral materials, thermally activated zeolite was prepared as follows:

The 5A zeolite powder was placed in a porcelain boat and the powder content was not more than two-thirds of the volume of the porcelain boat. The porcelain boat was placed in a muffle furnace with a program room temperature of 20 °C and a heating rate of 5 °C/min. The thermal activation temperature gradient was designed as shown in Table 2, in which the ZT group was the control group dried at 50 °C for 72 h. After holding for 2 h and cooling to room temperature, modified 5A zeolite as a raw material for the subsequent preparation was obtained.

#### 2.2.3. Preparation of Modified 5A Zeolite–Metakaolin Geopolymer

**Figure 2:**
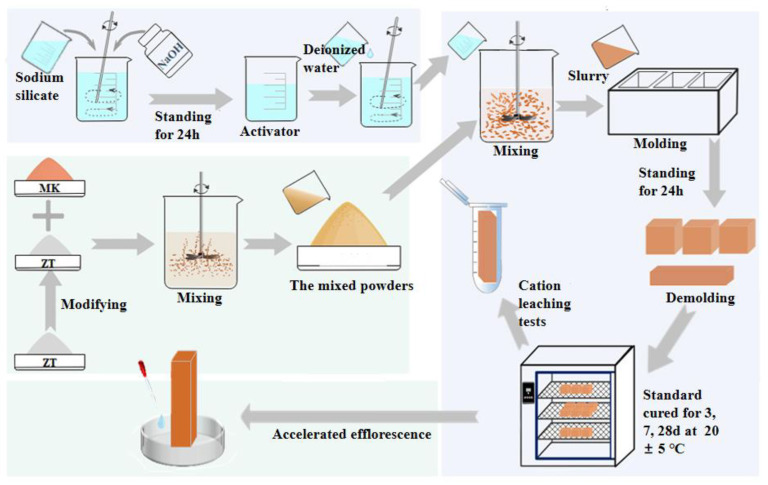
The preparation of modified-zeolite-based metakaolin geopolymer.

The mix proportion is listed in Table 3. The mass fraction of modified 5A zeolite and MK was 100%. Zeolite was mixed through internal mixing, while the activator and deionized water were added through external mixing. After being mixed and stirred well according to the ratio, the paste was transferred to 4 × 4 × 4 cm^3^ and 2 × 2 × 8 cm^3^ molds and cured at room temperature for 24 h. Then, the specimens were demolded and placed in a standard curing chamber under 20 °C and 95% relative humidity for a different number of curing days for mechanical properties tests, cation leaching tests, and accelerated efflorescence tests. Figure 2 provided a description of the geopolymer preparation. 

#### 2.2.4. Assessment of Anti-Efflorescence Performance of Geopolymers

Based on the methods from the relevant literature [23], the anti-efflorescence property was assessed in two steps:

1. After curing for 28 day, the samples were sealed in a large centrifuge tube and immersed in 40 mL deionized water for 7 day. Next, 10 mL of the top layer was removed and then filtered through a microporous membrane (0.45 µm). The pH was adjusted to the test range using 2% HNO_3_ and placed in a 25 mL volumetric flask. The corresponding dilution rates were calculated for the ICP test. And the leaching concentration of cations reflected the degree of efflorescence in the geopolymer.
2. To accelerate efflorescence, the samples cured for 28 day were immersed in a Petri dish with a water level of 2 mm and exposed to ambient air at 20 ± 5 °C, 50 ± 15% RH for 28 days. The initial water level was maintained by adding water daily, and physical photos of the specimens were taken after 28 days. Each sample had five surfaces where efflorescence products could be observed (the surface submerged in water was difficult to detect). Image Pro Plus software 6.0 was used to accurately quantify the visual degree of efflorescence by calculating the percentage of all efflorescence areas on the surface of each specimen. And the result is the average of three times of calculation.

### 2.3. Analysis and Testing

XRF analysis of the chemical composition of raw materials was carried out by using a PANalytical Axios X-ray fluorescence spectrometer (Malvern PANalytical, Almelo, The Netherlands). Microscopic images were provided by a scanning electron microscope model 20050089sb FEI, manufactured by FEI (Hong Kong) Ltd. (Hong Kong, China). The samples were freeze-dried for 24 h before testing and sprayed with gold. The raw material was analyzed using a Malvern Mastersizer 2000/3000 laser particle size analyzer (Malvern Instruments Ltd., Malvern, UK). The parameter of size range was set from 0.02 to 2000 μm with a stirrer speed of 1000 samples/s. The compressive strength of the sample cube with a size of 50 × 50 × 50 mm^3^ was tested using a universal testing machine. And the strength was determined based on the average value of three samples. The ICP-OES (inductively coupled plasma optical emission spectroscopy) test was conducted using the Optima 5300 DV (PerkinElmer, Shelton, CT, USA) to analyze the Na^+^ and Ca^2+^ concentrations in the leachate. And the linear range could reach 10^5^–10^6^ orders of magnitude. The surface area and pore structure of the samples were assessed from N_2_ adsorption/desorption isotherms using an ASAP 2460 (Micromeritics Instruments Corporation, GA, USA) surface area and porosimetry analyzer, and the flow rate of N_2_ was 80 mL/min.

## 3. Results and Discussion

### 3.1. Effect of Modification Temperature on Cation-Exchange Property of 5A Zeolite

**Figure 3:**
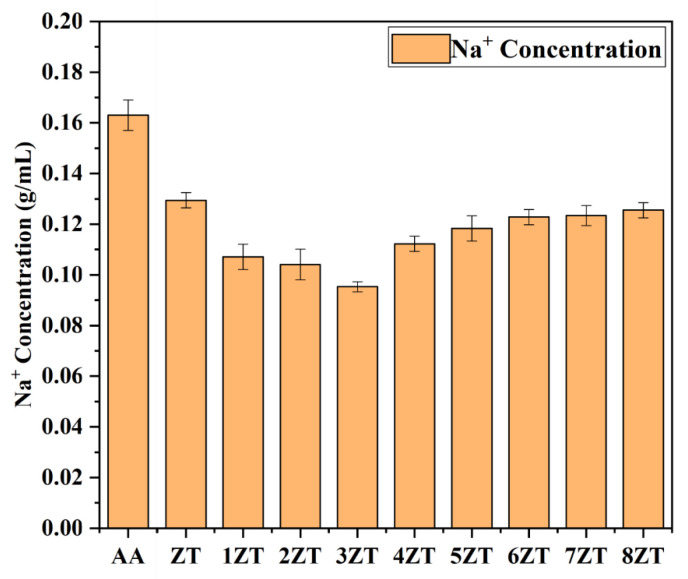
The Na^+^ adsorption of 5A zeolite in an alkaline condition.

Figure 3 shows the trend of Na^+^ adsorption on 5A zeolite in a sodium hydroxide solution at different thermal activation temperatures to explore the optimal modification temperature in alkaline conditions. As the thermal activation temperature increased, the Na^+^ concentration in solution tended to decrease and then increase. Firstly, the presence of a large number of cavities and pores provided the zeolite with a large specific surface area. The thermal activation temperature in the range of 100 and 300 °C could remove the adsorbed water and zeolite water in the pores, which facilitated an increase in the specific surface area and improved the adsorption and cation-exchange properties of the zeolite. Thermally activated zeolites and metal cations have a better water affinity [24]; this was macroscopically manifested as a decrease in the Na^+^ concentration in solution. However, the adsorption properties of zeolite are also related to its structure [25]. Excessive temperature led to changes in the internal structure of zeolite, with a decrease in the pore area and in the number of cavities on surface. At the same time, the breaking of chemical bonds inside zeolite led to a deterioration in the ion-exchange capacity of zeolite [26]. Above 300 °C, partial hydroxyl groups and bound water in zeolite were removed, which is the reason for the decrease in Na^+^ adsorption on zeolite to the same concentration of sodium hydroxide solution as the thermal activation temperature increases. Therefore, the thermal activation temperature of 300 °C was determined to be the optimal modification temperature, and 3ZT had an optimal cation-exchange property.

### 3.2. Basic Properties and Microstructure of Geopolymers

#### 3.2.1. Analysis of the Compressive Strength of Geopolymers

**Figure 4:**
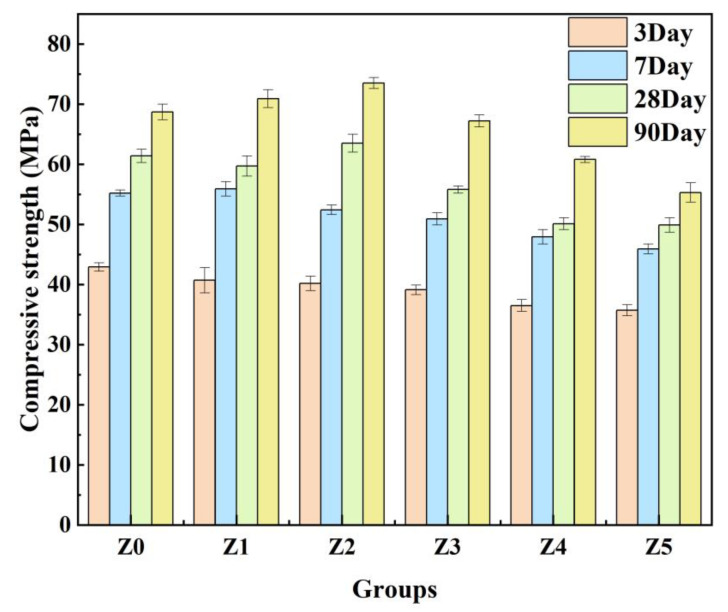
Effect of different modified-zeolite contents on the compressive strength of geopolymers.

Figure 4 shows the effect of 3ZT content on the compressive strength of the geopolymer. As can be seen from Figure 4, the compressive strength of samples cured for 3 days decreased slightly as the amount of 3ZT increased. Compared to the control group Z0, the compressive strength of the geopolymer decreased by 5.1%, 6.3%, 8.9%, 14.9%, and 16.8% in sequence as 3ZT replaced MK in equal amounts in 2 wt.% increments. This phenomenon can be ascribed to the early lower pozzolanic activity of ZT crystals and the water–air exchange effect of the pores formed in the zeolite during mixing with the fresh slurry [25].

As the curing time increased, the geopolymer with an appropriate 3ZT admixture gradually showed strength advantages. Compared to the control group Z0, the compressive strength of group Z1 increased by 1.3% and 4.7% at 7 and 90 days, respectively. The compressive strength of group Z2 increased by 3.4% and 7% at 28 and 90 days, respectively, while groups Z3–Z5 still showed a continuous decrease in strength. This means that an appropriate amount of 3ZT is beneficial to the strength of the geopolymer, and excessive content of 3ZT leads to a deterioration in strength. There are several possible reasons for such a trend:

1. Zeolites with a specific surface area of 1.1 m^2^/g can be distributed as micro-aggregates in the matrix to fill the pores of specimen, which can make the structure tightly packed and cause the specimen to be compacted [27].
2. Due to the water absorption of 3ZT, a moderate addition of 3ZT can cause water deficit in partial microregions and increase the alkalinity of the solution. A higher alkali content promotes the dissolution of active amorphous silica-alumina materials in MK and subsequently promotes the geopolymerization reaction [28]. This may be one of the reasons for the formation of a more homogeneous microstructure. In addition, the cavities and pores in 5A zeolite can absorb and desorb water [29]. The 5A zeolite can exchange water effectively with its surroundings because of this property and its porous structure. Under the water deficit condition, the zeolite water present in the pores and cavities will be gradually released with the extension of curing time, providing basic conditions for a continuous geopolymerization reaction to achieve an internal curing effect, which is macroscopically manifested by an enhancement in the compressive strength of specimens with an appropriate 3ZT content.
3. Temuujin et al. [30] showed that the addition of Ca^2+^ led to a more homogeneous and denser microstructure of the material. Yip et al. [31] suggested that the Ca(OH)_2_ formed by Ca^2+^ in an alkaline environment could provide additional nucleation sites for the binder system to form (N,C)-A-S-H gels and then promote the rapid formation of geopolymer gels. Combined with the microstructure of each curing stage, it is also evident that 3ZT has a cation-exchange effect in the system, which can absorb the alkali metal Na^+^ from the matrix and gradually diffuse Ca^2+^ into the surroundings. In the early stage of the reaction, the geopolymerization reaction is imperfect, with fewer oligomers depolymerized by the zeolite. The exchanged Ca^2+^ forms Ca(OH)_2_ in an alkaline environment [31]. As the geopolymerization proceeds, the number of oligomers gradually increases. The solubility product of the gel is much smaller than that of Ca(OH)_2_, resulting in a decrease in Ca^2+^ concentration in the matrix and the continuous dissolution of Ca(OH)_2_ crystals [32]. The low Ca^2+^ concentration is not sufficient to form C-S-H but may form (N,C)-A-S-H gels and may be immobilized as charge-balancing cations captured by surrounding N-A-S-H gels. During this process, excess metal cations are immobilized, and gels are formed to optimize the pore space. It is one of the reasons why an appropriate admixture of 3ZT enhances the strength [30,33].

An excessive admixture of 3ZT leads to a reduction in compressive strength. On the one hand, it may be that the free water in solution is adsorbed by the 5A zeolite, which reduces the fluidity of fresh cement slurry and increases the possibility of the introduction of defects into specimens. It may also be that the combined effects of agglomeration and water–air exchange result in the formation of a discontinuous structure and more defects to affect the mechanical properties of specimens. On the other hand, zeolite agglomerates during mixing. The peripheral zeolites absorb water and form a water film on the surface to prevent the internal zeolites from absorbing water. And the pozzolanic activity of synthetic zeolite 3ZT is much less than that of MK and natural zeolites [34]. Therefore, when the 3ZT content is too high, the increase in defects and the agglomeration of zeolites will lead to the formation of peripheral water films, which will affect the hydration process in the system.

#### 3.2.2. Analysis of the Pore Structure of Geopolymers

**Figure 5:**
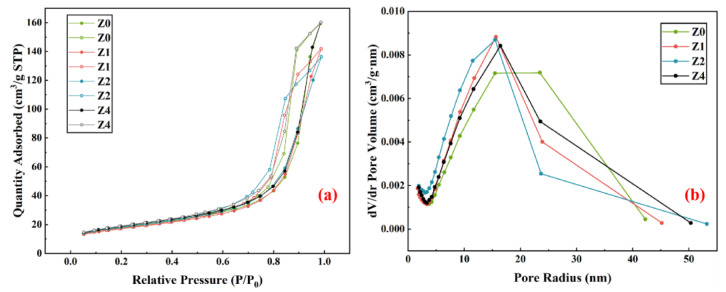
Effect of different modified-zeolite contents on the pore structure of geopolymers: (**a**) BET surface areas of geopolymers, (**b**) BJH analysis of pore size distribution.

Figure 5a shows that group Z1, with an appropriate amount of 3ZT, had a lower adsorption capacity and specific surface area, while the Z2 group showed lesser N_2_ adsorption and a larger specific surface area. This may be due to the addition of 3ZT forming smaller and more numerous pores in the matrix. Figure 5b shows that the critical pore size of the specimens shifts toward a smaller range with the addition of 3ZT. The number of mesopores of the Z2 curve in the 2–10 nm range is more than that in the other groups. The pore size can be generally classified as gel pore and capillary pore within the research field. And pores with a pore size above 20 nm are considered to be capillary pores, which are mainly responsible for transporting harmful substances such as sulfate attack ions and heavy metal ions [35,36]. The denser structure may be related to the micro-aggregate effect, the internal curing effect, and the formation of (N, C)-A-S-H gels in 3ZT [37]. In general, the larger the cumulative pore volume, the more severe the strength loss of the specimen [38]. This also microscopically explains the detrimental mechanical properties of geopolymers with a high admixture of 3ZT.

As shown in Table 4, the average pore size of the specimens increased first and then decreased with the increase in 3ZT content, which had a great influence on the microstructure of the geopolymer. The order of the porosity size was Z4 > Z0, while the order of the average pore size was Z0 > Z4. This result can be explained using the pore size distribution curve. The mesopore volume of the Z0 group was larger than that of Z4 in the 20–30 nm pore size range, while the mesopore volume of Z0 was smaller than that of Z4 in the >40 nm pore size range. This causes the average pore size of Z0 to move to higher values and also indicates that the addition of 3ZT facilitates the refinement of mesopores in the 20–30 nm pore size range. Meanwhile, a high content of 3ZT tends to form large pores, which is related to the interaction between zeolite and geopolymers [39].

#### 3.2.3. Microstructure Analysis of Geopolymers

**Figure 6:**
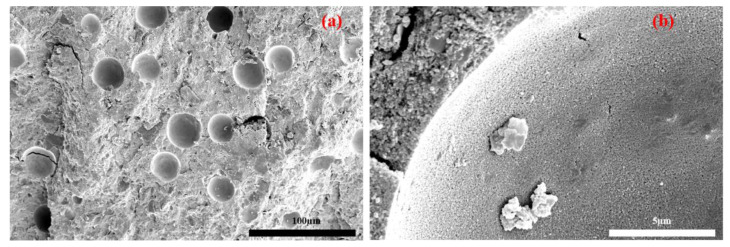
Microstructure of group Z2 after curing for 3 days: (**a**) large-scale microstructure, (**b**) characteristic microstructure.

The microstructure characteristics of the Z5 group after curing for 3 days are shown in Figure 6a,b. It can be seen from Figure 6a that samples of the Z5 group contain huge bubbles, which exist as defects in the matrix. Under external loads, cracks are easily derived from defects to reduce the strength of geopolymer [40]. This phenomenon is mainly related to the fact that a high amount of 3ZT deteriorates the fluidity of the cement paste and causes bubbles to be more difficult to discharge; then, the bubbles become defects that remain in the system. It can also be observed that some 5A zeolite agglomerates are present in many bubble pores. The water–air exchange effect of 5A zeolite tends to provide a nucleation site for small bubbles to aggregate in the matrix and gradually evolves them into large bubbles during matrix coagulation. As can be seen in Figure 6b, the early zeolite surface forms fine gel particles by cation exchange.

**Figure 7:**
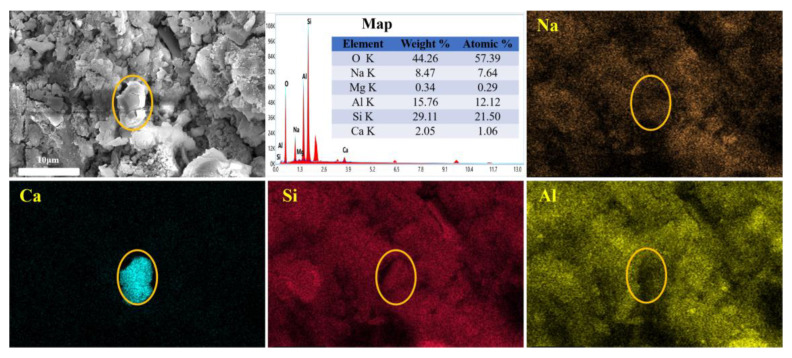
Characteristic microstructure of group Z2 after curing for 3 days.

EDS surface scanning was used to analyze the microregion components. Figure 7 clearly shows that early 3ZT is present in a relatively homogeneous state and is not well embedded within the surrounding matrix. This is probably due to the water–air exchange of 3ZT, which forms an air film around the zeolite and prevents the migration of the gel to the zeolite [26]. It also demonstrates the low reactivity of 3ZT in the early stage compared to MK. At the same time, the distortion of cubic zeolites is probably due to the cation exchange of 5A zeolites through the solution. Ca^2+^ migrates to the surface of the zeolite and easily forms Ca(OH)_2_ attached to the surface under alkaline conditions [41].

**Figure 8:**
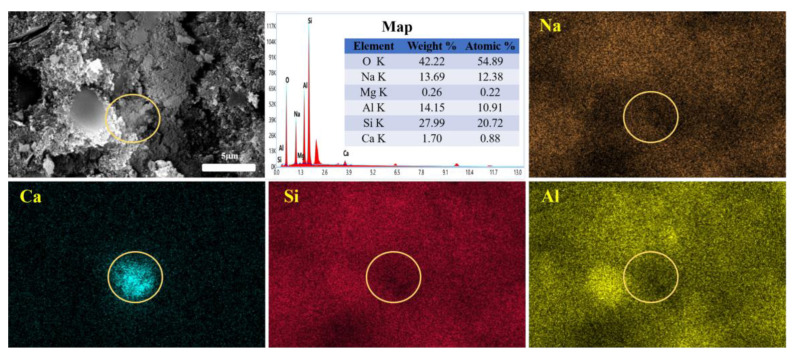
Characteristic microstructure of group Z2 after curing for 90 days.

The microstructure of group Z2 after curing for 90 days is shown in Figure 8. It can be seen that 3ZT is completely surrounded and well embedded within the hydration products in the matrix, with no obvious interfacial transition zones. The multicavity structure enables 3ZT to absorb and desorb water effectively from the surroundings, which also provides the basic conditions for a continuous geopolymerization reaction to obtain an internal curing effect. The intensity of each element in the EDS surface scan shows that the main elements are Si, Al, O, Na, and Ca. The aggregation of Na and Ca is observed in the microregion, and Ca shows a tendency to spread in all directions. This indicates that Na^+^ in the matrix has been exchanged with Ca^2+^ in the zeolite, with some of the Na^+^ exchanged to the interior of the zeolite, while the replaced Ca^2+^ diffuses around the 5A zeolite to form gels. However, the interactions between calcium, silicates, and aluminates are complex [42], and many factors influence the formation of hydration products. Many scholars have suggested that when silicon-alumina-rich metakaolin is used as the raw material, the hydration product is (N, C)-A-S-H with a three-dimensional network structure [43,44]. Thus, the tight interfacial binding between the zeolites and the hydration products may be the result of the formation of (N, C)-A-S-H gels after cation exchange and the internal curing effect in 5A zeolites [45,46].

### 3.3. The Property of Anti-Efflorescence of Geopolymers

**Figure 9:**
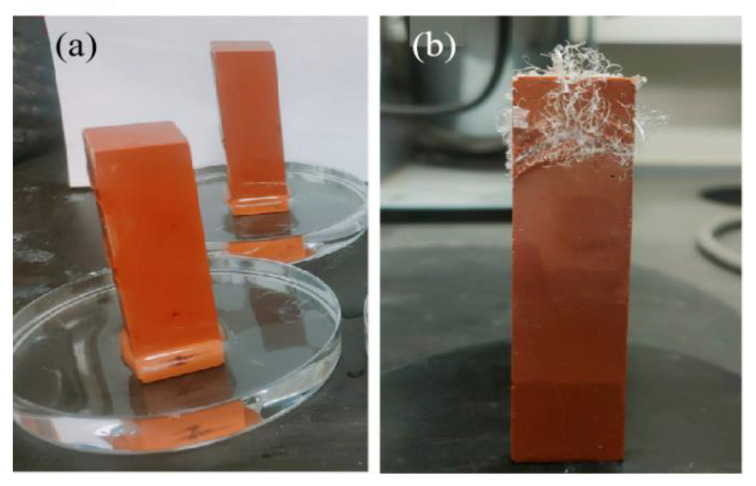
Physical picture of specimen: (**a**) simulated accelerated efflorescence condition, (**b**) efflorescent white precipitate on the surface.

The white carbonate precipitate produced on the surface of specimens is a visual intuitive phenomenon of efflorescence, which can initially indicate the degree of efflorescence in geopolymer. Figure 9 shows the physical picture of a specimen under a simulated accelerated efflorescence condition. The specimens were exposed to a simulated accelerated efflorescence condition for 28 days and the phenomenon of efflorescence was evident.

**Figure 10:**
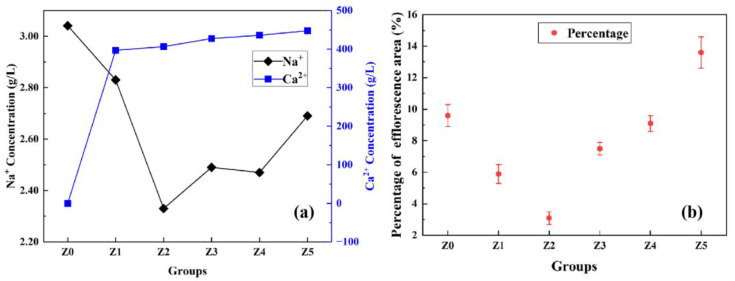
Evaluation of efflorescence resistance: (**a**) cation leaching, (**b**) visual efflorescence area percentage.

The Na^+^ and Ca^2+^ concentrations in the leachate of geopolymer with different 3ZT contents are shown in Figure 10a. With an increase in the 3ZT content, the Ca^2+^ concentration in the solution increased but leveled off later, while the Na^+^ concentration decreased rapidly. After the Na^+^ concentration reached the lowest point at 4 wt.% of 3ZT with a 19.4% reduction in leaching, as the 3ZT content continued to increase, the Na^+^ concentration showed an increasing trend. 

This is due to the property of 3ZT to exchange Ca^2+^ and Na^+^ with its surroundings. And a large number of broken chemical bonds on the surface of MK particles undergo geopolymerization reaction under an alkaline condition [47]. Compared to group Z0, the addition of 3ZT contributes to a reduction in the free Na^+^ in the system [48]. The quantitative analysis of Na^+^ concentration in the leachate demonstrates that the cation-exchange property of the geopolymer facilitates a decrease in the free alkali metal content in the system to reduce the degree of efflorescence. However, the Na^+^ concentration in the solution tends to increase at a later stage. This may be due to the fact that some 3ZT agglomerates with the increasing content. The gel structure in the matrix becomes discontinuous; thus, more defects are introduced into the specimen. This results in a high content of unfixed Na^+^ in the pore solution, which leaches into the solution through the connected pores in the specimen.

The Image Pro Plus image processing software is often used to quantify images to visually understand the description given by pixels [49,50]. The degree of visual efflorescence was assessed using the IPP software, and the statistical efflorescence area percentage of the specimen is shown in Figure 10b. It can be seen that compared with group Z0, group Z2 (3ZT mixed at 4 wt.%) had the lowest degree of visual efflorescence and the efflorescence area of the specimens was reduced by 57.3%, which indicates that a 4 wt.% dosage of 3ZT can effectively inhibit efflorescence in geopolymers. Moreover, with a high amount 3ZT, the efflorescence was obvious. When the content of 3ZT was 10 wt.%, the efflorescence area of the specimen increased by 9.4%, which is related to the free Na+ concentration in specimens and the pore structure in cementitious materials. A suitable amount of 3ZT can act as particles filling in the matrix and optimizing the pores [27]. As 3ZT exchanges cations in the matrix, the concentration of free Na^+^ is reduced, and the exchanged Ca^2+^ is captured as an equilibrium charge to form (N, C)-A-S-H gels. The internal curing effect of 3ZT can make the geopolymerization reaction more complete [28]. It reduces the concentration of excess free Na^+^ in the matrix and forms denser gels to fill the voids. Less free Na^+^ participates in the acid–base neutralization reaction, and the reaction produces less efflorescence products, which is macroscopically manifested as a low degree of efflorescence. When the 3ZT admixture is too high, the agglomeration of 5A zeolites tends to reduce the fluidity of the cement slurry and introduce a large number of defects into the system. At the same time, the reduction in the amount of highly reactive MK in the system leads to the formation of discontinuous gels, making the matrix to react with external CO_2_ through the connected pores to form carbonates and show obvious efflorescence phenomena easily.

## 4. Conclusions

1. The water–air exchange of zeolites can provide nucleation conditions for bubbles, which can introduce defects that are detrimental to the early compressive strength of the geopolymer. An appropriate amount of 3ZT facilitates the enhancement of compressive strength at a later stage, and the optimal content was 4 wt.%. Compared to the MK geopolymer (group Z0), the compressive strength of samples in group Z2 increased by 3.4% and 7% at 28 and 90 days, respectively.
2. The appropriate amount of 3ZT facilitates the geopolymerization reaction and the formation of more gel pores to reduce the average pore size. This is probably the result of the synergistic effect of 3ZT as a micro-aggregate, internal curing, and cation exchange in the matrix.
3. The cation exchange of 3ZT in the matrix is effective in reducing the Na^+^ concentration and the degree of efflorescence in the pore solution. The Z2 group (3ZT dosed at 4 wt.%) performed best with a 19.4% reduction in Na^+^ leaching and a 57.3% reduction in efflorescence.
4. The cation-exchange pattern of 3ZT in the matrix is as follows: In the cement slurry, the previous thermal activation modification reduces the resistance of water films on the zeolite surface. Then, 3ZT adsorbs part of the hydrated Na^+^ in exchange for a part of the Ca^2+^ to balance the charge. With the dissolution and reorganization of the surrounding matrix, Ca^2+^ exists as Ca(OH)_2_ in the alkaline environment. As the matrix gradually gelates, the highly soluble Ca(OH)_2_ gradually dissolves to release Ca^2+^, and the matrix is captured to form (N, C)-A-S-H gels, making 5A zeolite embed well within the hydration products. This process both fixes excess free Na^+^ in the matrix and optimizes the pore size distribution.

## Figures and Tables

**Table 1:** Composition of raw materials/wt.%.

Oxide	ZT	MK
Na_2_O	5.29	0.57
MgO	-	0.9
Al_2_O_3_	37.05	41.94
SiO_2_	42.24	53.73
P_2_O_5_	-	0.06
SO_3_	0.13	0.37
K_2_O	0.63	0.05
CaO	14.25	0.54
Fe_2_O_3_	0.03	1.55
LOI	0.38	0.29

**Table 2:** Thermal activation temperature of different groups of 5A zeolite.

Sample No.	Heating Rate/°C/min	Thermal Activation Temperature/°C	Holding Time/h
ZT	0	50	72
1ZT	5	100	2
2ZT	5	200	2
3ZT	5	300	2
4ZT	5	400	2
5ZT	5	500	2
6ZT	5	600	2
7ZT	5	700	2
8ZT	5	800	2

**Table 3:** Mix proportion of geopolymer.

Sample No.	Modified Zeolite/wt.%	MK/wt.%	Alkali Activator/wt.%	Water/wt.%
Z0	0	100	90	10
Z1	2	98	90	10
Z2	4	96	90	10
Z3	6	94	90	10
Z4	8	92	90	10
Z5	10	90	90	10

**Table 4:** Average pore size of modified 5A zeolite–metakaolin geopolymers.

Group	Z0	Z1	Z2	Z4
Average pore size/nm	13.8611	12.1878	11.0179	13.1219

## Data Availability

Data are contained within the article.
